# Taming diffusion models for image restoration: a review

**DOI:** 10.1098/rsta.2024.0358

**Published:** 2025-06-19

**Authors:** Ziwei Luo, Fredrik Gustafsson, Zheng Zhao, Jens Sjölund, Thomas Schön

**Affiliations:** ^1^ Department of Information Technology, Uppsala University, Uppsala, Sweden; ^2^ Department of Medical Epidemiology and Biostatistics, Karolinska Institutet, Stockholm, Sweden; ^3^ Department of Computer and Information Science, Linköping University, Linköping, Sweden

**Keywords:** Diffusion model, image restoration, Generative models, inverse problems

## Abstract

Diffusion models (DMs) have achieved remarkable progress in generative modelling, particularly in enhancing image quality to conform to human preferences. Recently, these models have also been applied to low-level computer vision for photo-realistic image restoration (IR) in tasks such as image denoising, deblurring and dehazing. In this review, we introduce key constructions in DMs and survey contemporary techniques that make use of DMs in solving general IR tasks. We also point out the main challenges and limitations of existing diffusion-based IR frameworks and provide potential directions for future work.

This article is part of the theme issue ‘Generative modelling meets Bayesian inference: a new paradigm for inverse problems’.

## 1. Introduction

**Figure 1.**
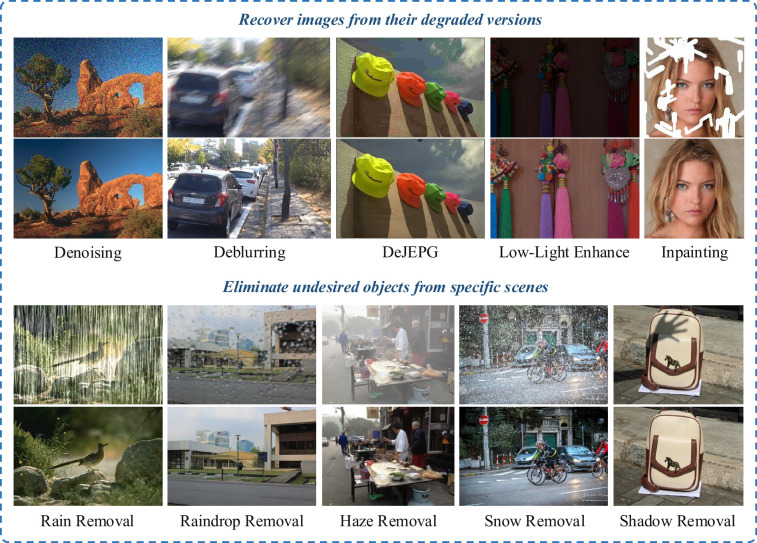
Generally, there are two types of IR tasks: (1) Recover images from their degraded versions and (2) eliminate undesired objects from specific scenes. Here, all top rows are LQ input images and the bottom rows are the corresponding HQ images generated by a diffusion-based IR model [5]. As observed, applying DMs for IR can produce photo-realistic results in line with human perceptual preferences.

Image restoration (IR) is a long-standing and challenging research topic in computer vision, which generally has two high-level aims: (i) recover high-quality (HQ) images from their degraded low-quality (LQ) counterparts, and (ii) eliminate undesired objects from specific scenes. The former includes tasks like image denoising [1] and deblurring [2], while the latter contains tasks like rain/haze/snow removal [3] and shadow removal [4]. Figure 1 showcases examples of these applications. To solve different IR problems, traditional methods require task-specific knowledge to model the degradation and perform restoration in the spatial or frequency domain, by combining classical signal processing algorithms [6–8] with specific image-degradation parameters [9]. More recently, numerous efforts have been made to train deep learning models on collected datasets to improve performance on different IR tasks [10]. Most of them directly train neural networks on sets of paired LQ–HQ images with a reconstruction objective (e.g. $\ell_{1}$ or $\ell_{2}$ distances) as typical in supervised learning. While effective, this approach tends to produce over-smooth results, particularly in textures [11]. Although this issue can be alleviated by including adversarial or perceptual losses [12], the training then typically becomes unstable and the results often contain undesired artefacts or are inconsistent with the input LQ images [11].

Recently, generative diffusion models (DMs) [13] have drawn increasing attention due to their stable training process and remarkable performance in producing realistic images and videos [14]. Inspired by them, numerous works have incorporated the diffusion process into various IR problems to obtain high-perceptual/photo-realistic results [15–18]. However, these methods exhibit considerable diversity and complexity across various domains and IR tasks, obscuring the shared foundations that are key to understanding and improving diffusion-based IR approaches. In light of this, our paper reviews the key concepts in DMs and then surveys trending techniques for applying them to IR tasks. More specifically, the fundamentals of DMs are introduced in §2, in which we further elucidate the score-based stochastic differential equations (Score-SDEs) and then show the connections between denoising diffusion probabilistic models (DDPMs) and Score-SDEs. In addition, the conditional diffusion models (CDMs) are elaborated such that we can learn to guide the image generation, which is key in adapting DMs for general IR tasks. Several diffusion-based IR frameworks are then summarized in §3. In particular, we can leverage CDMs for IR from different perspectives including DDPMs, Score-SDE and their connections. The connection even yields a training-free approach for non-blind IR, i.e. for tasks with known degradation parameters. Finally, we conclude the paper with a discussion of the remaining challenges and potential future work in §4.

## 2. Generative modelling with DMs

Generative DMs are a family of probabilistic models employing an iterative process (e.g. Markov chains) to transform the data distribution into a reference distribution. In the following, §2a describes a typical formulation of DMs: the DDPMs [13,19], followed by §2b which generalizes this to Score-SDEs for a more detailed analysis of the diffusion/reverse process. Finally, in §2c, we further show how to guide DMs for conditional generation, which is a key enabling technique for diffusion-based IR.

### (a) DDPMs

**Figure 2.**
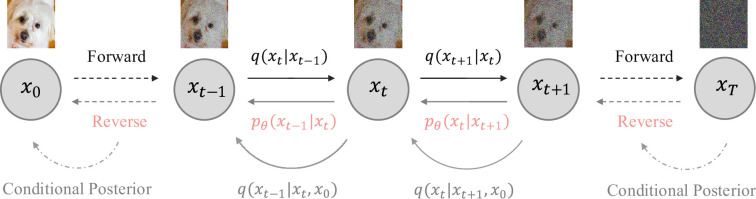
DDPMs. The forward path transfers data to Gaussian noise, and the reverse path learns to generate data from noise along the actual time reversal of the forward process. Here, the reverse transition distribution $p_{\theta }(x_{t-1}|x_{t})$ represents the model we aim to learn, and the conditional posterior $q(x_{t-1}|x_{t},x_{0})$ is a tractable Gaussian which serves as the target distribution the model wants to match as the $L_{t}$ term in equation (2.7).

Given a variable $x_{0}$ sampled from a data distribution $p_{0}(x)$ , DDPMs [13,19] are latent variable models consisting of two Markov chains: a forward/diffusion process $q(x_{1:T}|x_{0})$ and a reverse process $p_{\theta }(x_{0:T})$ . The forward process transfers $x_{0}$ to a Gaussian distribution by sequentially injecting noise. For simplicity, we set *$q_{0}(x)=p_{0}(x)$* such that the forward process starts from the data distribution. Then the reverse process learns to generate new data samples starting from the Gaussian noise. An overview of the DDPM is shown in figure 2. Below, we explain the forward and backward processes, and provide details on how the DDPMs are trained.

#### (i) Forward diffusion process

The forward process perturbs data samples $x_{0}$ to noise $x_{T}$ . It can be characterized by a joint distribution encompassing all intermediate states, represented in the form


(2.1) $$\begin{array}{lcr} & q(x_{1:T}|x_{0})=\prod_{t=1}^{T}q(x_{t}|x_{t-1}),\;x_{0}∼p_{0}(x). & \end{array}$$

Here, the transition kernel $q(x_{t}|x_{t-1})$ is a handcrafted Gaussian given by


(2.2) $$\begin{array}{lcr} & q(x_{t}|x_{t-1})=N(x_{t};\sqrt{1-\beta_{t}}x_{t-1},\beta_{t}I), & \end{array}$$

where $\beta_{1:T}\in \left(0,1\right)$ is the variance schedule: a set of pre-defined hyper-parameters that ensure the forward process (approximately) converges to a Gaussian distribution. Let $\alpha_{t}=1-\beta_{t}$ and ${\bar{\alpha }}_{t}=\prod_{s=1}^{t}\alpha_{s}$ , equation (2.2) then allows us to marginalize the joint distribution of equation (2.1) to


(2.3) $$\begin{array}{lcr} & q(x_{t}|x_{0})=N(x_{t};\sqrt{{\bar{\alpha }}_{t}}x_{0},(1-{\bar{\alpha }}_{t})I). & \end{array}$$

We usually set $\beta_{1}<\beta_{2}<\cdots <\beta_{T}$ such that $\alpha_{1}>\alpha_{2}>\cdots >\alpha_{T}\approx 0$ and the terminal distribution $q(x_{T})\approx N(x_{T};0,I)$ is thus a standard Gaussian, which allows us to generate new data points by reversing the diffusion process starting from sampled Gaussian noise. Moreover, it is important to note that posteriors along the forward process are tractable when conditioned on the original data sample $x_{0}$ , i.e. $q(x_{t-1}|x_{t},x_{0})$ is a tractable Gaussian [19]. This tractability enables the derivation of the DDPM training objective, which we will describe in §2a(iii).

#### (ii) Reverse process

In contrast, the reverse process learns to match the actual time reversal of the forward process, which is also a joint distribution modelled by $p_{\theta }(x_{0:T})$ as follows:


(2.4) $$\begin{array}{lcr} & p_{\theta }(x_{0:T})=p(x_{T})\prod_{t=1}^{T}p_{\theta }(x_{t-1}|x_{t}),\;x_{T}∼N(0,I). & \end{array}$$

In DDPMs, the transition kernel $p_{\theta }(x_{t-1}|x_{t})$ is defined as a learnable Gaussian:


(2.5) $$\begin{array}{lcr} & p_{\theta }(x_{t-1}|x_{t})=N(x_{t-1};\mu_{\theta }(x_{t},t),\;\Sigma_{\theta }(x_{t},t)I), & \end{array}$$

where $\mu_{\theta }$ and $\Sigma_{\theta }$ are the parameterized mean and variance, respectively. Learning the latent variable model of equation (2.5) is key to DDPMs since it substantially affects the quality of data sampling. That is, we have to adjust the parameters θ until the final sampled variable $x_{0}$ is close to that sampled from the real data distribution.

#### (iii) Training objective

To learn the reverse process, we usually minimize the variational bound on the negative log-likelihood which introduces the forward joint distribution of equation (2.1) in the objective L as


(2.6) $$\begin{array}{r} E_{q_{0}(x)}[-\log p_{\theta }(x_{0})]\leq \underset{\text{negative evidence lower bound }}{\underbrace{E_{q(x_{0:T})}[-\log\frac{p_{\theta }(x_{0:T})}{q(x_{1:T}|x_{0})}]}}=E_{q}[-\log p(x_{T})-\sum_{t=1}^{T}\log\frac{p_{\theta }(x_{t-1}|x_{t})}{q(x_{t}|x_{t-1})}]. \end{array}$$

Here, $p(x_{T})$ is a standard Gaussian, $p_{\theta }(x_{t-1}|x_{t})$ is the reverse transition kernel in equation (2.5) that we want to learn, and $q(x_{t}|x_{t-1})$ is the forward transition kernel of equation (2.2). This objective can be further rewritten according to


(2.7) $$\begin{array}{r} L:=E_{q}[\underset{L_{T}}{\underbrace{D_{KL}(q(x_{T}|x_{0})||p(x_{T}))}}+\sum_{t=2}^{T}\underset{L_{t-1}}{\underbrace{D_{KL}(q(x_{t-1}|x_{t},x_{0})||p_{\theta }(x_{t-1}|x_{t}))}}\underset{L_{0}}{\underbrace{-\log p_{\theta }(x_{0}|x_{1})}}], \end{array}$$

where $L_{T}$ is called the prior matching term and contains no learnable parameters, $L_{t-1}$ is the posterior matching term and $L_{0}$ the data reconstruction term that maximizes the likelihood of $x_{0}$ . Sohl-Dickstein *et al*. [19] prove that the conditional posterior distribution in $L_{t-1}$ is a tractable Gaussian: $q(x_{t-1}|x_{t},x_{0})=N(x_{t-1};{\tilde{\mu }}_{t}(x_{t},x_{0}),{\tilde{\beta }}_{t}I)$ , where the mean and variance are


(2.8) $${\tilde{\mu }}_{t}(x_{t},x_{0})=\frac{\sqrt{\alpha_{t}}(1-{\bar{\alpha }}_{t-1})}{1-{\bar{\alpha }}_{t}}x_{t}+\frac{\sqrt{{\bar{\alpha }}_{t-1}}\beta_{t}}{1-{\bar{\alpha }}_{t}}x_{0},\;\text{and}\;{\tilde{\beta }}_{t}=\frac{1-{\bar{\alpha }}_{t-1}}{1-{\bar{\alpha }}_{t}}\beta_{t}.$$

All terms in *${\tilde{\beta }}_{t}$* are known and thus the posterior variance in equation (2.5) can be non‐parametric, i.e. *$\Sigma_{\theta }(x_{t},t)={\tilde{\beta }}_{t}$ *, which does not depend on *$x_{t}$* and allows us to only focus on learning the posterior mean *$\mu_{\theta }(x_{t},t)$* . Specifically, applying the reparameterization trick to $q(x_{t}|x_{0})$ of equation (2.3) gives an estimate of the initial state: $x_{0}=\frac{1}{\sqrt{{\bar{\alpha }}_{t}}}(x_{t}-\sqrt{1-{\bar{\alpha }}_{t}}\epsilon_{t})$ , which can be substituted into equation (2.8) to obtain: ${\tilde{\mu }}_{t}(x_{t},x_{0})=\frac{1}{\sqrt{\alpha_{t}}}(x_{t}-\frac{1-\alpha_{t}}{\sqrt{1-{\bar{\alpha }}_{t}}}\epsilon_{t})$ . The noise $\epsilon_{t}$ then can be learned using a neural network $\epsilon_{\theta }(x_{t},t)$ , and the parameterized distribution mean can be rewritten as


(2.9) $$\begin{array}{lcr} & \mu_{\theta }(x_{t},t)=\frac{1}{{\sqrt{\alpha }}_{t}}(x_{t}-\frac{1-\alpha_{t}}{\sqrt{1-{\bar{\alpha }}_{t}}}\epsilon_{\theta }(x_{t},t)). & \end{array}$$

The transition kernel $p_{\theta }(x_{t-1}|x_{t})$ of equation (2.5) is finally updated according to the following:


(2.10) $$\begin{array}{lcr} & p_{\theta }(x_{t-1}|x_{t})=N(x_{t-1};\mu_{\theta }(x_{t},t),\;{\tilde{\beta }}_{t}I), & \end{array}$$

where the variance ${\tilde{\beta }}_{t}$ is predefined as in equation (2.8). Note that, $p_{\theta }(x_{t-1}|x_{t})$ now matches the form of $q(x_{t-1}|x_{t},x_{0})=N(x_{t-1};{\tilde{\mu }}_{t}(x_{t},x_{0}),{\tilde{\beta }}_{t}I)$ , to minimize the KL term of $L_{t-1}$ in equation (2.7). Also note that DDPMs only need to learn the noise network $\epsilon_{\theta }(x_{t},t)$ , for which it is common to use a U-Net architecture with several self-attention layers [13]. The noise network $\epsilon_{\theta }(x_{t},t)$ takes an image $x_{t}$ and a time t as input, and outputs a noise image of the same shape as $x_{t}$ . More specifically, the scalar time t is encoded into vectors similar to positional embedding [20] and is combined with $x_{t}$ in the feature space for time-varying noise prediction.

#### Simplified objective

We now have known expressions for all components of the objective L in equation (2.7). However, its current form is not ideal to use for model training since it requires $L_{t}$ to be computed at every timestep of the entire diffusion process, which is time-consuming and impractical. Fortunately, the prior matching term $L_{T}$ can be ignored since it does not contain any parameters. By substituting equations (2.8) and (2.9) into equation (2.7), we also find that the final expanded version of the posterior matching term $L_{t-1}$ ( t∈{2,…,T} ) and the data reconstruction term $L_{0}$ have similar forms,


(2.11) $$\begin{array}{lcr} & L_{t-1}=\frac{\beta_{t}}{2\alpha_{t}(1-{\bar{\alpha }}_{t-1})}E_{x_{0},\epsilon }[\|\epsilon_{t}-\epsilon_{\theta }(x_{t},t){\|}^{2}]\;\mathrm{and}\;L_{0}=\frac{1}{2\alpha_{1}}E_{x_{0},\epsilon }[\|\epsilon_{1}-\epsilon_{\theta }(x_{1},1){\|}^{2}], & \end{array}$$

where *$E_{x_{0},\epsilon }$ *denotes *$E_{x_{0}∼q_{0}(x),\epsilon ∼N(0,I)}$* . By ignoring the weights outside the expectations in equation (2.11), the final training objective can therefore be obtained according to the following [13]:


(2.12) $$\begin{array}{r} L_{\text{simple}}:=E_{x_{0},t,\epsilon }[\|\epsilon_{t}-\epsilon_{\theta }(x_{t},t){\|}^{2}]=E_{x_{0},t,\epsilon }[\|\epsilon_{t}-\epsilon_{\theta }(\sqrt{{\bar{\alpha }}_{t}}x_{0}+\sqrt{1-{\bar{\alpha }}_{t}}\cdot \epsilon ,t){\|}^{2}], \end{array}$$

which essentially learns to match the predicted and real added noise for each training sample and thus is also called the *noise matching loss*. Compared to the original objective L in equation (2.7), $L_{\text{simple}}$ is a re-weighted version that puts more focus on larger timesteps t , which empirically has been shown to improve the training [13]. Once trained, the noise prediction network $\epsilon_{\theta }(x_{t},t)$ can be used to generate new data $x_{0}$ by running equation (2.10) starting from $x_{T}∼N(0,I)$ , i.e. by iterating


(2.13) $$\begin{array}{lcr} & x_{t-1}=\mu_{\theta }(x_{t},t)+\sqrt{{\tilde{\beta }}_{t}}\epsilon \;\mathrm{where}\;\mu_{\theta }(x_{t},t)=\frac{1}{\sqrt{\alpha_{t}}}(x_{t}-\frac{1-\alpha_{t}}{\sqrt{1-{\bar{\alpha }}_{t}}}\epsilon_{\theta }(x_{t},t)), & \end{array}$$

as a parameterized data sampling process, similar to that in Langevin dynamics [21].

### (b) Data perturbation and sampling with SDEs

**Figure 3.**
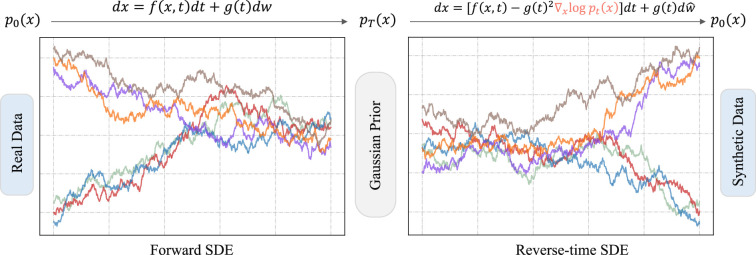
Data perturbation and sampling with SDEs. In contrast to DDPMs, the Score-SDE continuously perturbs the data to Gaussian noise using a forward SDE, dx=f(x,t)dt+g(t)dw , and then generates new samples by estimating the score $\nabla_{x}\log\;p_{t}(x)$ and simulating the corresponding reverse-time SDE.

We have shown how DDPM works for data perturbation and data generation. We can further generalize the DDPM to stochastic differential equations, namely, Score-SDE [22], where both the forward and reverse processes are in continuous-time state spaces. This generalization offers a deeper insight into the mathematics behind DMs that underlies the success of diffusion-based generative modelling. Figure 3 shows an overview of the Score-SDE approach.

#### (i) Data perturbation with forward SDEs

Here, we construct variables $\{x(t){\}}_{t\in [0,T]}$ for data perturbation in continuous time, which can be modelled as a forward SDE defined by


(2.14) $$\begin{array}{lcr} & \;dx=f(x,t)\;dt+g(t)\;dw,\;x(0)∼p_{0}(x), & \end{array}$$

where f(x,t) and g(t) are called the *drift* and *diffusion* functions, respectively, and w is a standard Wiener process (also known as Brownian motion). We use $p_{t}(x)$ to denote the marginal probability density of x(t) , and use p(x(t)|x(s)) to denote the transition kernel from x(s) to x(t) . Moreover, we always design the SDE to drift to a fixed prior distribution (e.g. standard Gaussian), ensuring that x(T) becomes independent of $p_{0}(x)$ and can be sampled individually.

#### (ii) Sampling with reverse-time SDEs

We can sample noise and reverse the forward SDE to generate new data close to that sampled from the real data distribution. Note that reversing equation (2.14) yields another diffusion process, i.e. a reverse-time SDE [23] in the form


(2.15) $$\begin{array}{lcr} & \;dx=[f(x,t)-g(t{)}^{2}\;\nabla_{x}\log\;p_{t}(x)]\;dt+g(t)\;d\hat{w},\;x(T)∼p_{T}(x), & \end{array}$$

where $\hat{w}$ is a reverse-time Wiener process and $\nabla_{x}\log\;p_{t}(x)$ is called the score (or score function). The score $\nabla_{x}\log\;p_{t}(x)$ is the vector field of x pointing to the directions in which the probability density function has the largest growth rate [21]. Once the score is known for all time t , simulating equation (2.15) in time allows us to sample new data from noise.

Earlier work such as the score-based generative models [21] often learn the score using *score matching* [24]. However, score matching is computationally costly and only works for discrete times. Song *et al*. [22] propose a continuous-time version that optimizes the following:


(2.16) $$\begin{array}{lcr} & E_{t,x(0),x(t)}[\|s_{\theta }(x(t),t)-\nabla_{x(t)}\log\;p_{t}(x(t)\;|\;x(0)){\|}^{2}], & \end{array}$$

where t is uniformly sampled over [0,T] , $x(0)∼p_{0}(x)$ , $x(t)∼p_{t}(x(t)|x(0))$ , and $s_{\theta }(x(t),t)$ represents the score prediction network. This objective ensures that the optimal score network, denoted $s_{\theta }^{*}(x(t),t)$ , from equation (2.16) satisfies $s_{\theta }^{*}(x(t),t)=\nabla_{x}\log\;p_{t}(x)$ almost surely [22,25].

#### (iii) Interpreting DDPM with the variance preserving SDE

Notably, extending DDPM to an infinite number of timesteps (i.e. continuous timesteps) leads to a special SDE which gives a more reliable interpretation of the diffusion process, and allows us to optimize the sampling with more efficient SDE/ordinary differential equation (ODE) solvers [22,26]. Specifically, recall the DDPM perturbation kernel $q(x_{t}|x_{t-1})$ of equation (2.2) and write it in the form


(2.17) $$x_{i}=\sqrt{1-\beta_{t}}\;x_{i-1}+\sqrt{\beta_{i}}\;\epsilon_{i-1},\;\epsilon ∼N(0,I)\;\;\text{and}\;\;i=1,\ldots ,N,$$

where i is the discrete timestep. Let us define an auxiliary set $\{{\bar{\beta }}_{i}=N\beta_{t}{\}}_{i=1}^{N}$ and obtain


(2.18) $$\begin{array}{lcr} & x_{i}=\sqrt{1-\frac{{\bar{\beta }}_{i}}{N}}\;x_{i-1}+\sqrt{\frac{{\bar{\beta }}_{i}}{N}}\;\epsilon_{i-1}. & \end{array}$$

As a preparation to convert functions from discrete-time to continuous-time, let $\beta (\frac{i}{N}):={\bar{\beta }}_{i}$ , $x(\frac{i}{N}):=x_{i}$ and $\epsilon (\frac{i}{N}):=\epsilon_{i}$ . We can now rewrite equation (2.18) with the difference $\Delta t=\frac{1}{N}$ and time $t\in 0,\frac{1}{N},\ldots ,\frac{N-1}{N}$ as follows:


(2.19)x(t+Δt)=1−β(t+Δt)Δtx(t)+β(t+Δt)Δtϵ(t)(2.20)≈x(t)−12β(t)Δtx(t)+β(t+Δt)Δtϵ(t)(Taylor series)(2.21)≈x(t)−12β(t)Δtx(t)+β(t)Δtϵ(t),

where the two approximate equalities hold when Δt→0 . Then we convert Δt to dt , $\sqrt{\Delta t}\;\epsilon (t)$ to dw and obtain the following: $\;dx=-\frac{1}{2}\beta (t)x\;dt+\sqrt{\beta (t)}\;dw$ , which is a typical mean-reverting SDE (also known as the Ornstein–Uhlenbeck process [27]) that drifts towards a stationary distribution, i.e. a standard Gaussian in this case. Song *et al*. [22] also name it the variance preserving SDE (VP-SDE) and further illustrate that DDPM’s marginal distribution $q(x_{t}|x_{0})$ in equation (2.3) is a solution to the VP-SDE. Therefore, we can use either the diffusion reverse process (equation 2.13) or the reverse-time SDE (equation 2.15) to sample new data from noise with the same trained DDPM. In addition, the score $\nabla_{x}\log\;p_{t}(x)$ can be directly computed from the marginal distribution $q(x_{t}|x_{0})$ in equation (2.3),


(2.22) $$\begin{array}{lcr} & \nabla_{x_{t}}\log\;p_{t}(x_{t})=-\frac{x_{t}-\sqrt{{\bar{\alpha }}_{t}}x_{0}}{\left(1-{\bar{\alpha }}_{t}\right)}=-\frac{\epsilon_{t}}{\sqrt{1-{\bar{\alpha }}_{t}}}, & \end{array}$$

where $\epsilon_{t}$ is from the reparameterization trick and can be approximated using the noise prediction network $\epsilon_{\theta }(x_{t},t)$ . Equation (2.22) thus shows how we convert the DM to an SDE (i.e. obtain the score $\nabla_{x}\log\;p_{t}(x)$ from noise $\epsilon_{\theta }(x_{t},t)$ ). Then, numerous efficient SDE/ODE solvers can be used to optimize DMs, further bringing interpretability and faster sampling [22].

### (c) CDMs

So far, we have learned how to sample data from different types of DMs. However, all of the above methods are concerned with unconditional generation, which is insufficient for IR where we want to sample HQ images conditioned on degraded LQ images. Therefore, we present the CDM below.

Let us keep the diffusion process $q(x_{1:T}|x_{0})$ of equation (2.1) unchanged and reconstruct the reverse process in equation (2.4) with a condition y , i.e. $p_{\theta }(x_{0:T}|y)=p(x_{T}|y)\prod_{t=1}^{T}p_{\theta }(x_{t-1}|x_{t},\;y)$ . The conditional reverse kernel can then be modelled as


(2.23) $$\begin{array}{lrr} & p_{\theta ,\phi }(x_{t-1}|x_{t},\;y)=Z\cdot p_{\theta }(x_{t-1}|x_{t})\;p_{\phi }(y|x_{t-1}), & \end{array}$$

where $p_{\phi }(y|x)$ is an additional network that predicts y from x , and $Z=p_{\phi }(y|x_{t}{)}^{-1}$ can be treated as a constant since it does not depend on $x_{t-1}$ . This equation yields an adjusted mean for the posterior distribution of equation (2.10), given by [28]


(2.24) $$\begin{array}{lcr} & {\hat{\mu }}_{\theta }(x_{t},t,y)=\mu_{\theta }(x_{t},t)+\eta \cdot {\tilde{\beta }}_{t}\nabla_{x_{t}}\log\;p_{\phi }(y|x_{t}), & \end{array}$$

where η is the gradient scale (also called the guidance scale). Equation (2.22) further gives the score of the joint distribution $p_{t}(x_{t},y)$ :


(2.25)∇xtlog⁡pt(xt,y)=∇xtlog⁡pt(xt)+∇xtlog⁡pt(y|xt)(2.26)≈−11−α¯t(ϵθ(xt,t)−1−α¯t∇xtlog⁡pϕ(y|xt)),

which provides a conditional noise predictor ${\hat{\epsilon }}_{\theta }$ with the following form [28]


(2.27) $$\begin{array}{lcr} & {\hat{\epsilon }}_{\theta }(x_{t},t,y)=\epsilon_{\theta }(x_{t},t)-\eta \cdot \sqrt{1-{\bar{\alpha }}_{t}}\nabla_{x_{t}}\log\;p_{\phi }(y\;|\;x_{t}). & \end{array}$$

The conditional sampling is performed as a regular DDPM by substituting this new noise predictor ${\hat{\epsilon }}_{\theta }(x_{t},t,y)$ into the posterior mean of equation (2.9). The gradient scale η controls the performance trade-off between image quality and fidelity, i.e. lower η produces photo-realistic results, and higher η yields better consistency with the condition.

#### (i) Conditional SDE

Similar to guided diffusion, we can also change the score function to control the reverse-time SDE conditioned on the variable y , i.e. by replacing $\nabla_{x}\log\;p_{t}(x)$ with $\nabla_{x}\log\;p_{t}(x\;|\;y)$ in equation (2.15). Since $p_{t}(x\;|\;y)\propto p_{t}(x)\;p_{t}(y\;|\;x)$ , the conditional score can be decomposed as


(2.28) $$\begin{array}{lcr} & \nabla_{x}\log\;p_{t}(x\;|\;y)=\nabla_{x}\log\;p_{t}(x)+\nabla_{x}\log\;p_{t}(y\;|\;x), & \end{array}$$

which means that we can simulate the following reverse-time SDE for conditional generation:


(2.29) $$\begin{array}{lcr} & \;dx=[f(x,t)-g(t{)}^{2}\;(\nabla_{x}\log\;p_{t}(x)+\nabla_{x}\log\;p_{t}(y\;|\;x))]\;dt+g(t)\;d\hat{w}, & \end{array}$$

where $x(T)∼p_{T}(x\;|\;y)$ . Song *et al*. [22] show that we can use a separate network to learn $p_{t}(y\;|\;x)$ (e.g. a time-dependent classifier if y represents class labels), or estimate its log gradient $\nabla_{x}\log\;p_{t}(y\;|\;x)$ directly with heuristics and domain knowledge.

With these CDMs, we can sample images with specified labels (such as dog and cat) or, as the main topic of this paper, recover clean HQ images from corrupted LQ inputs.

## 3. DMs for IR

Diffusion-based IR can be considered a special case of CDMs with image conditioning. We first introduce the concept of image degradation, which is a process that transforms an HQ image x into an LQ image y characterized by undesired corruptions. The general image degradation process can be modelled as follows:


(3.1) $$\begin{array}{lcr} & y=A(x)+n, & \end{array}$$

where A denotes the degradation function and n is additive noise. As the examples show in figure 1, degradation can manifest itself in various forms such as noise, blur, rain and haze. IR then aims to reverse this process to obtain a clean HQ image from the corrupted LQ counterpart y .

IR is further decomposed into two distinct settings, *blind and non-blind IR*, depending on whether or not the degradation parameters A and n in equation (3.1) are known. *Blind IR* is the most general setting, in which no explicit knowledge of the degradation process is assumed. Blind IR methods instead utilize datasets of paired LQ–HQ images for supervised training of models. *Non-blind IR* methods, in contrast, assume access to A and n . This is an unrealistic assumption for many important real-world IR tasks, and thus limits non-blind methods to a subset of specific IR tasks such as bicubic downsampling, Gaussian blurring, colourization, or inpainting with a fixed mask. In the following, we first describe the most straightforward diffusion-based approach for general *blind* IR tasks in §3a. Representative *non-blind* diffusion-based approaches are then covered in §3b. Finally, §3c covers more recent methods for general *blind* IR.

### (a) Conditional direct DM

The most straightforward approach for applying DMs to general IR tasks is to use the CDM with image guidance from §2c. In the IR context, the term $p_{\phi }(y|x)$ in equation (2.27) represents the image degradation model which can be either a fixed operator with known parameters or a learnable neural network, depending on the task. It is also noted that strong guidance (large η in equation 2.27) leads to good fidelity but visually LQ results (e.g. over-smooth images), while weak guidance (small η ) has the opposite effect [28]. Now, let us consider the extreme case: *how about decreasing η to zero, i.e. no guidance?* A simple observation from equation (2.27) is that with η=0 , the conditional noise predictor learns the unconditional noise predictor directly: ${\hat{\epsilon }}_{\theta }(x_{t},t,y)=\epsilon_{\theta }(x_{t},t)$ , and the objective for diffusion-based IR is given by


(3.2) $$\begin{array}{lcr} & L_{\mathrm{cddm}}=E_{x_{0},y,t,\epsilon }[\|\epsilon_{t}-{\hat{\epsilon }}_{\theta }(\sqrt{{\bar{\alpha }}_{t}}x_{0}+\sqrt{1-{\bar{\alpha }}_{t}}\cdot \epsilon ,t,y){\|}^{2}]. & \end{array}$$

**Figure 4.**
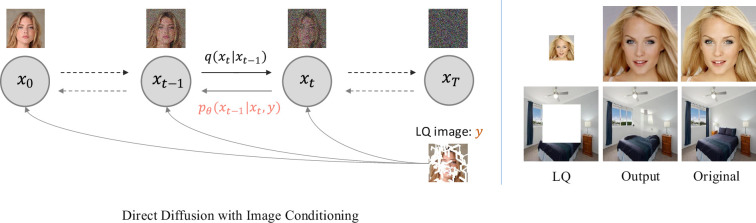
*Left*: Overview of CDDM on the face inpainting case. The only change compared to DDPM (figure 2) is the reverse transition model $p_{\theta }(x_{t-1}|x_{t},\;y)$ , which involves the LQ image y in sampling to generate the corresponding HQ image. *Right*: Two IR examples (image super-resolution and inpainting) performed under the CDDM framework. These results look realistic but are not consistent with the original image.

We name this the conditional direct diffusion model (CDDM), which essentially follows the same training and sampling procedure as DDPM, except for the condition y in the noise prediction as shown in figure 4. As a result, the generated image can be of very high visual quality (it looks realistic), but often has limited consistency with the original HQ image [16], as can be observed for the examples in the right-hand part of figure 4. Fortunately, some IR tasks, such as image super-resolution, colourization and inpainting, are highly ill-posed and can tolerate diverse predictions. CDDM can then be effectively applied to these tasks for photo-realistic IR.

One typical method is SR3 [16], which employs CDDM with a few modifications for image super-resolution. To condition the model on the LQ image y , SR3 up-samples y to the target resolution so that y can be concatenated with the intermediate state $x_{t}$ along the channel dimension. Subsequently, Palette [29] extends SR3 to general IR tasks including colourization, inpainting, uncropping and JPEG restoration. There is more work [17,30] employing the same ‘direct diffusion’ strategy but adopting different restoration pipelines and additional networks for task-specific model learning. More recently, Wang *et al*. [31] propose StableSR, which further adapts a large-scale pretrained DM (Stable Diffusion [14]) for IR, by tweaking the noise predictor with image conditioning in the same way as for CDDM.

### (b) Training-free CDMs

The key to the success of CDDM in IR lies in learning the conditional noise predictor ${\hat{\epsilon }}_{\theta }(x_{t},t,y)$ by optimizing equation (3.2) on a dataset of paired LQ–HQ images. Unfortunately, this means that ${\hat{\epsilon }}_{\theta }(x_{t},t,y)$ needs to be re-trained to handle tasks which are not included in the current training data, even in the non-blind setting where the degradation parameters A and n in equation (3.1) are known. For non-blind IR, a *training-free* approach can instead be derived by directly incorporating the degradation function into a pretrained unconditional DM, such as a DDPM.

With known degradation parameters, the term p(y|x) also becomes accessible: $p(y|x)=N(A(x),\sigma_{n}^{2}I)$ , if the noise n is Gaussian. Traditional IR approaches often solve this problem using *maximum a posteriori* (MAP) estimation [6], as follows:


(3.3) $$\begin{array}{lcr} & \hat{x}=\arg\;\min_{x}\;\frac{1}{2\sigma_{n}^{2}}\|y-A(x){\|}^{2}+\lambda P(x), & \end{array}$$

where P(x) is a prior term empirically chosen to characterize the prior knowledge of x . Then, a natural idea is to incorporate a pretrained unconditional DDPM into P(x) as a powerful learned image prior. Specifically, recall the conditional score of equation (2.28) in the form


(3.4) $$\begin{array}{lcr} & \nabla_{x_{t}}\log\;p_{t}(x_{t}\;|\;y)=\nabla_{x_{t}}\log\;p_{t}(x_{t})+\nabla_{x_{t}}\log\;p_{t}(y\;|\;x_{t}), & \end{array}$$

where $x_{t}$ matches the diffusion state in DDPM, and the unconditional score $\nabla_{x_{t}}\log\;p_{t}(x_{t})$ can be obtained from equation (2.22) and approximated with DDPM’s noise predictor, as $\nabla_{x_{t}}\log\;p_{t}(x_{t})\approx s_{\theta }(x_{t},t)=-\frac{\epsilon_{\theta }(x_{t},t)}{\sqrt{1-{\bar{\alpha }}_{t}}}$ . However, computing $p_{t}(y|x_{t})$ in equation (3.4) is difficult since there is no obvious relationship between y and the state $x_{t}$ . Fortunately, with Gaussian noise $n∼N(0,\sigma_{n}^{2}I)$ , Chung *et al*. [32] propose an approximation for $\nabla_{x_{t}}\log\;p_{t}(y\;|\;x_{t})$ at each timestep t :


(3.5) $$\begin{array}{lcr} & \nabla_{x_{t}}\log\;p_{t}(y\;|\;x_{t})\approx \nabla_{x_{t}}\log\;p_{t}(y\;|\;{\hat{x}}_{0}),\;\mathrm{where}\;{\hat{x}}_{0}=\frac{1}{\sqrt{{\bar{\alpha }}_{t}}}(x_{t}+(1-{\bar{\alpha }}_{t})s_{\theta }(x_{t},t)). & \end{array}$$

This can be obtained via Tweedie’s formula [32–35]. The approximation above is motivated by a Dirac approximation *$\nabla_{x_{t}}\log\;p(y|x_{t})=\nabla_{x_{t}}\log\;\int p(y|x_{0})\;p(x_{0}|x_{t})dx_{0}\approx \nabla_{x_{t}}\log\;p(y|{\hat{x}}_{0}(x_{t}))$ *, where *${\hat{x}}_{0}(x_{t})$* is any Monte Carlo sample of *$p(x_{0}|x_{t})$ *. However, the approximation usually exhibits high variance since it uses a single Monte Carlo sample, while drawing more samples incurs more computations. Moreover, it is worth noting that $p_{t}(y|{\hat{x}}_{0})$ is a tractable Gaussian: $p_{t}(y|{\hat{x}}_{0})=N(A({\hat{x}}_{0}),\sigma_{n}^{2}I)$ . Computing $\nabla_{x_{t}}\log\;p_{t}(y\;|\;{\hat{x}}_{0})$ and substituting it for $\nabla_{x_{t}}\log\;p_{t}(y\;|\;x_{t})$ in equation (3.4) thus gives the following:


(3.6) $$\begin{array}{lcr} & \nabla_{x_{t}}\log\;p_{t}(x_{t}\;|\;y)\approx s_{\theta }(x_{t},t)-\frac{1}{2\sigma_{n}^{2}}\nabla_{x_{t}}\|y-A({\hat{x}}_{0}){\|}^{2}. & \end{array}$$

We can then incorporate this approximation equation (3.6) into the sampling of a pretrained DDPM,


(3.7)xt−1=1α¯t(xt+(1−αt)∇xtlog⁡pt(xt|y))+β~tϵ(3.8)≈1α¯t(xt+(1−αt)[sθ(xt,t)−12σn2∇xt‖y−A(x^0)‖2])+β~tϵ(3.9)=−1−αt2σn2α¯t∇xt‖y−A(x^0)‖2⏟data consistency term+1α¯t(xt+(1−αt)sθ(xt,t))+β~tϵ⏟diffusion term,

where the first line is derived from equations (2.13) and (2.22) with additional condition y . Note that the diffusion term in equation (3.9) is actually an unconditional sampling step in DDPM, where $s_{\theta }$ is obtained from equation (2.22) as $s_{\theta }(x_{t},t)=-\frac{\epsilon_{\theta }(x_{t},t)}{\sqrt{1-{\bar{\alpha }}_{t}}}$ . By letting $\rho =\frac{1-\alpha_{t}}{2\sigma_{n}^{2}\sqrt{{\bar{\alpha }}_{t}}}$ represent the step size of the data consistency term and simplifying the diffusion term, we then finally have


(3.10) $$\begin{array}{lcr} & x_{t-1}=-\rho \nabla_{x_{t}}\|y-A({\hat{x}}_{0}){\|}^{2}+\mu_{\theta }(x_{t},t)+\sqrt{{\tilde{\beta }}_{t}}\;\epsilon , & \end{array}$$

where $\mu_{\theta }$ and $\tilde{\beta }$ are the posterior mean and variance of equation (2.10), respectively. This approach is called the diffusion posterior sampling (DPS) [32,36]. Note that equation (3.10) is conceptually similar to the MAP estimate in equation (3.3), with $\nabla_{x_{t}}\|y-A({\hat{x}}_{0}){\|}^{2}$ as the data consistency term and $\mu_{\theta }(x_{t},t)+\sqrt{{\tilde{\beta }}_{t}}\;\epsilon$ being a diffusion-based image prior. When the degradation parameters of equation (3.1) are known, DPS thus utilizes this knowledge to guide the sampling process of a pretrained DDPM, encouraging generated images to be consistent with the LQ input image y .

**Figure 5.**
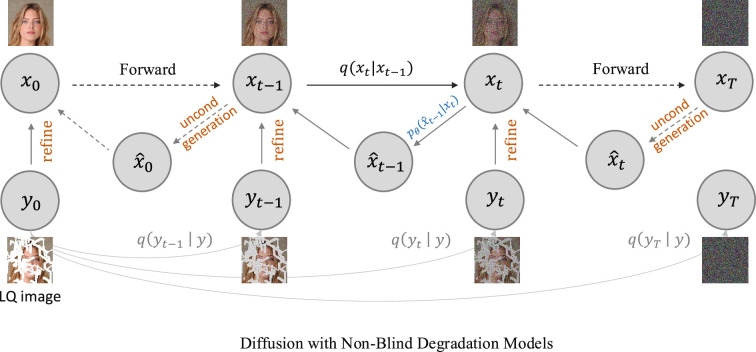
Overview of the projection-based CDM. There are two paths for the HQ image x and LQ image y , generated from the same DM. At each reverse step t , the sampling first leverages the pretrained DDPM for unconditional generation, i.e. $p_{\theta }({\hat{x}}_{t}|x_{t+1})$ , and then refines ${\hat{x}}_{t}$ to $x_{t}$ with functions H and b as $x_{t}=H({\hat{x}}_{t})+b(y_{t})$ , where $y_{t}$ is obtained by applying the forward marginal transition equation (2.3) on the LQ image as $y_{t}∼q(y_{t}|y)$ .

However, DPS does rely on the approximation in equation (3.5), for which the approximation error approaches zero only when the noise n of y has a high variance: $\sigma_{n}\to \infty$ . For the case where the LQ image is noiseless, y=A(x) , we would prefer to introduce the approach from figure 5 where the unconditional generated state ${\hat{x}}_{t}$ is refined using the known degradation A and the LQ image y . More specifically, since the term $\nabla_{x_{t}}\log\;p_{t}(y\;|\;x_{t})$ now is unattainable (or non-approximable), we instead apply the forward marginal transition of equation (2.3) also on the LQ image y to obtain $y_{t}∼q(y_{t}|y)=N(y_{t};\sqrt{{\bar{\alpha }}_{t}}y,(1-{\bar{\alpha }}_{t})I)$ , which is an intermediate state between y and Gaussian noise. Then, we impose data consistency by projecting ${\hat{x}}_{t}$ onto a conditional path as follows:


(3.11) $$\begin{array}{lcr} & x_{t}=H({\hat{x}}_{t})+b(y_{t}),\;\mathrm{where}\;{\hat{x}}_{t}=\mu_{\theta }(x_{t},t)+\sqrt{{\tilde{\beta }}_{t}}\;\epsilon , & \end{array}$$

where H and b are functions derived from the known degradation A . For computational efficiency, the two functions are typically assumed to be linear and tailored to specific IR tasks. This projection-based method is referred to as iterative latent variable refinement [37]. In addition, for linear degradation problems, we can refine the intermediate state *$x_{t}$* by decomposing *A* into partitions and then combine them with the LQ image *y* in the reverse process [15], or optimize *$x_{t}$* using the Bayesian framework directly [38]*.* These approaches are similar to the projection-based method but can be more computationally efficient.

Recently, another class of approaches for training-free CDMs have emerged which are based on Feynman–Kac models and sequential Monte Carlo (SMC) samplers [39–41]. At the core, they wrap the approximations for *$p_{t}(y|x_{t})$* in the proposals of an SMC sampler, so that the marginal distributions of the sampler anneals to the target conditional one. This approach is statistically exact, regardless of the approximations in *$p_{t}(y|x_{t})$ *, in the sense that as the number of particles used in the SMC sampler goes to infinity, the resulting population converges in distribution to the target. As such, this type of method improves significantly over DPS [32] in terms of statistical errors. However, it comes at the cost of storing a population of particles which does not scale well in memory in the problem dimension, and the efficiencies of their proposals. To improve the sampler, Cardoso *et al*. [40] consider linear Gaussian likelihood models and propose efficient proposals based on an inpainting problem, while Janati *et al*. [41] develop a divide-and-conquer construction to set intermediate target distributions. Note that although Corenflos *et al*. [42], Dou & Song [43] also use SMC samplers, they target different Feynman–Kac models. Moreover, the methods in [42] are training-free only for special problems (e.g., inpainting).

### (c) Diffusion process towards degraded images

**Figure 6.**
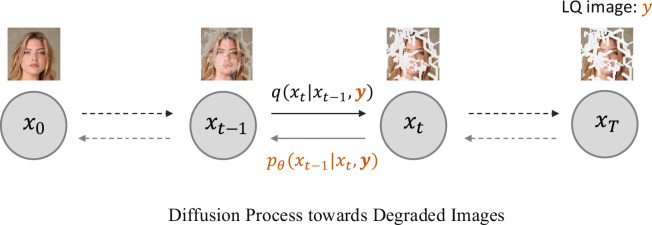
Overview of the approach that performs diffusion towards degraded images. Here, the LQ image y is involved in both the forward and backward processes. Moreover, the terminal state $x_{T}$ is a (noisy) LQ image rather than Gaussian noise.

In previous sections, we have presented several diffusion-based IR methods, both for the blind and non-blind settings. However, these methods all generate images starting from Gaussian noise, which intuitively should be inefficient for IR tasks, given that input LQ images are closely related to the corresponding HQ images. That is, it should be easier to translate directly from the LQ image to the HQ image, rather than from noise to HQ image, as shown in figure 6. To address this problem, for general blind IR tasks, Luo *et al*. [18] propose the IR-SDE that models image degradation with a mean-reverting SDE:


(3.12) $$\begin{array}{lcr} & dx=\theta_{t}\;(\mu -x)dt+\sigma_{t}dw, & \end{array}$$

where μ is the state mean the SDE drifts to. The parameters $\theta_{t}$ and $\sigma_{t}$ are predefined and they control the speed of the mean-reversion and the stochastic volatility, respectively. It is noted that the VP-SDE [22] is a special case of equation (3.12) where μ is set to 0. Moreover, the SDE in equation (3.12) is proven to be tractable when the coefficients satisfy $\sigma_{t}^{2}\;/\;\theta_{t}=2\;\lambda^{2}$ for all timesteps [18]. Similar to DDPM, we can obtain the marginal transition kernel $p_{t}(x)$ , which is a Gaussian given by


(3.13) $$\begin{array}{lcr} & p_{t}(x_{t}|x_{0})=N(x_{t};\;\mu +(x_{0}-\mu )\;e^{-{\bar{\theta }}_{t}},\;\lambda^{2}\;(1-e^{-2\;{\bar{\theta }}_{t}})), & \end{array}$$

where ${\bar{\theta }}_{t}=\int_{0}^{t}\theta_{z}\;dz$ . As t→∞ , the terminal distribution converges to a stationary Gaussian with mean μ and variance $\lambda^{2}$ . By setting the HQ image as the initial state $x_{0}$ and the LQ image as the terminal state mean μ , this SDE iteratively transforms the HQ image into the LQ image with additional noise (where the noise level is fixed to λ ). Then, we can restore the HQ image based on the reverse-time process of equation (3.12) as follows:


(3.14) $$dx=[\theta_{t}\;(\mu -x)-\sigma_{t}^{2}\;\nabla_{x}\log p_{t}(x)]dt+\sigma_{t}d\hat{w}.$$

Notably, the score function *$\nabla_{x}\log\;p_{t}(x)$* is tractable when conditioning on the known *$x_{0}$* in training, as *$\nabla_{x}\log\;p_{t}(x\;|\;x_{0})=-\frac{x_{t}-m_{t}}{v_{t}}$ *, where *$m_{t}$* and *$v_{t}$* are the mean and variance of equation (3.13), respectively. Learning this score with a neural network is similar to denoising score matching [25] but the target score is directly computed from the training distributions.

However, IR-SDE still needs to add noise to the LQ image as a terminal state $x_{T}$ . For fixed point-to-point mapping with a diffusion process, we further introduce the diffusion bridge (DB) [44] which can naturally transfer complex data distributions to reference distributions, i.e. directly from HQ to LQ images, without adding noise. More specifically, given a diffusion process defined by a forward SDE as in equation (2.14), Rogers & Williams [45] show that we can force the SDE to drift from the HQ image x to a particular condition (the LQ image y ) via Doob’s h -transform [46]:


(3.15) $$\begin{array}{lcr} & \;dx=f(x,t)\;dt+{g(t)}^{2}h(x_{t},t,y,T)+g(t)\;dw, & \end{array}$$

where $h(x_{t},t,y,T)=\nabla_{x_{t}}\log\;p(x_{T}\;|\;x_{t}){\;|\;}_{x_{T}=y}$ is the gradient of the log transition kernel from t to T , derived from the original SDE. By setting the terminal state $x_{T}=y$ , the term ${g(t)}^{2}h(x_{t},t,y,T)$ pushes each forward step towards the end condition y , which exactly models the image degradation process. The corresponding reverse-time SDE of equation (3.15) can then be written as


(3.16) $$\begin{array}{lcr} & \;dx=[f(x,t)-g(t{)}^{2}\;(s(x_{t},t,y,T)-h(x_{t},t,y,T))]\;dt+g(t)\;d\hat{w}, & \end{array}$$

where $s(x_{t},t,y,T)=\nabla_{x_{t}}\log\;p(x_{t}\;|\;x_{T}){\;|\;}_{x_{T}=y}$ is the conditional score function which can be learned via score-matching. The HQ image can then be recovered from the LQ image y by iteratively running equation (3.16) in time as a traditional SDE solver. Note that we can design specific SDEs (e.g., VP/VE-SDE [22] to make the function $h(x_{t},t,y,T)$ tractable [44,47,48]. The simplest case is the Brownian bridge [44] which constructs the marginal distribution as *$p(x_{t}|x_{0},x_{T})=N((1-\frac{t}{T})x_{0}+\frac{t}{T}x_{T},\frac{t(T-t)}{T}I)$ *. Another particular case is the Schrödinger bridge [48], which aims to compute a diffusion process that interpolates within the optimal coupling (when the reference measure of the bridge is chosen to be a Brownian motion) between the HQ and LQ image distributions [49]. The solution of the Schrödinger bridge converges weakly to an optimal transport plan with respect to 2-Wasserstein [48,50]. Most DB frameworks learn the noise *$\epsilon_{\theta }(x_{t},t)$* directly by adopting the similar score reparameterization trick from equation (2.22), which leads to the following objective: *$\|\epsilon_{\theta }(x_{t},t)-\frac{x_{t}-m_{t}}{\sqrt{v_{t}}}\|$ *, where *$m_{t}$* and *$v_{t}$* are the marginal mean and variance of the forward process. More recently, Yue *et al*. [51] further propose to apply the DB to IR-SDE as the generalized Ornstein-Uhlenbeck bridge to achieve better performance. However, designing the forward SDE in equation (3.15) with a tractable yet effective $h(x_{t},t,y,T)$ remains a challenge and is under-explored in IR. With the growing popularity of Score-SDEs and DBs, we hope that future approaches will offer various efficient and elegant solutions to general IR problems.

## 4. Conclusion and discussion

DMs have shown incredible capabilities and gained significant popularity in generative modelling. In particular, the mathematics behind them make these models exceedingly elegant. Building on their core concepts, we have described several approaches that effectively employ DMs for various IR tasks, achieving impressive results. However, it is also crucial to highlight the main challenges and further outline potential directions for future work.

### (a) Difficult to process out-of-distribution degradations

**Figure 7.**
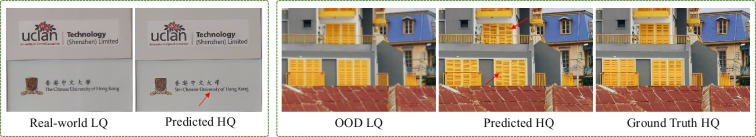
Failed examples of applying a trained DM [53] to real-world and OOD LQ input images. In the left-hand example, the predicted HQ image contains unrecognizable text. In the right-hand example, the generated window shutters are visually unpleasant and inconsistent with the LQ input image.

Applying the trained DMs to out-of-distribution (OOD) data often leads to inferior performance and produces visually unpleasant artifacts [52], as shown in figure 7. There is research [31] proposing to address this issue by introducing the capable stable diffusion [14] with a feature control module [54]. However, such approaches still have to refine the stable DM with specific IR datasets. Moreover, the commonly used synthetic data strategy [55] just simulates known degradations such as noise, blur and compression and is unable to cover all corruption types that might be encountered in real-world applications. Inspired by the success of large language models and vision-language models, more recent approaches [5,52,53] have begun to explore the use of various language-based image representations in IR. The main idea is to produce ‘clean’ text descriptions of input LQ images, describing the main image content without undesired degradation-related concepts, and use these to guide the restoration process.

### (b) Inconsistency in image generation

While DMs produce photo-realistic results, the generated details are often inconsistent with the original input, especially regarding texture and text information, as shown in the right-hand part of figure 4 and in figure 7. This is mainly due to the intrinsic bias in the multi-step noise/score estimation and the stochasticity of the noise injection in each iteration. One solution is to add a predictor to generate the initial HQ image (with $\ell_{1}$ loss) and then gradually add more details via a diffusion process [30]. However, this requires an additional network and the performance highly depends on the trained predictor. IR-SDE [18] proposes a maximum likelihood objective to learn the optimal restoration path, but its reverse-time process still contains noise injection (i.e. Wiener process) thus leading to unsatisfactory results. Recently, flow matching and optimal transport have shown great potential in image generation. In particular, they can form straight‐line trajectories in inference, which are more efficient than the curved paths from the DMs [56]. The use of such methods for IR tasks is therefore a seemingly promising future direction.

### (c) High computational cost and inference time

Most diffusion-based IR methods require a significant number of diffusion steps to generate the final HQ image (typically 1000 steps using DDPMs), which is both time-consuming and computationally costly, thus bringing challenges for deployment in various real-world applications. This problem can be alleviated using latent DMs [14,57] or efficient sampling techniques [26,58]. Unfortunately, these are not always suitable for IR tasks since the latent DM often produces colour shifting [31], and the efficient sampling would decrease the image generation quality [58]. Considering the particularity of IR, several works [18,42,48] design the diffusion process towards degraded images (§3c), such that their inference can start from the LQ image (rather than Gaussian noise). While this makes the sampling process more efficient (typically requiring less than 100 diffusion steps), it could be possible to improve further by designing more effective SDEs or DB functions.

#### (i) Closing

We have covered the basics of DMs and key techniques for applying them to IR tasks. This is an active research area with many interesting challenges and potential future directions, such as achieving photo-realistic yet consistent image generation, robustness to real-world image degradations, and more computationally efficient sampling. We hope that this review paper offers a foundational understanding that enables readers to gain deeper insights into the mathematical principles underlying advanced diffusion-based IR approaches.

## Data Availability

This article has no additional data.
